# Sympathy

**Published:** 1886-12-25

**Authors:** 


### WORDS OF CONSOLATION.

[Communications for this column should be addressed, "Chaplain," care of the Editor of THE HOSPITAL, who will gratefully welcome any suggestions or enquiries from matrons, nurses, and the staff generally.]

### XIII.—SYMPATHY.

"WE have not an high priest that cannot be touched with the feelings of our infirmities" (Heb. iv, 15). Why? Because His sufferings were so intense! Yes, but also because they were so various. "He was in all points tempted," tried, afflicted, like as we are. The sin of the world might, for ought we know, have been atoned for by death without such an awful complication of agony as our bl ssed Lord endured. But the variety of His manifold sufferings has given Him such an unique position as a Sympathizer, that he can say to every one of us, whatever the nature of our pain, "My child I can feel with you; I have borne your individual cross; I have known in my own bitter experience what you now feel." Here is a never failing source of comfort for the sick if they will only go to the fountain. Here is the fountain of all sympathy. For whatsoever we receive from others in this way, is but a vibration from the Head through the members. The sympathy of a fellow Christian is the mind of Christ produced through the Holy Spirit's working, and manifested towards us.

Consider for a moment the meaning of the word, and you will better understand why the truest sympathy is so rare, and why it should be sought for first of all in Christ. The word "sympathy" is a composition of two Greek words, which together signify "suffering *with*," or "feeling together with," some other person. Now this is clearly something more than merely feeling for others. We may be very sorry for a sufferer, and pity him, and speak kind words to him, and take a great deal of trouble to show the depth of our sincerity; but we do not, we cannot sympathise, in the strictest sense of the word, unless we have ourselves suffered somewhat in the same way. When we come to discuss our troubles with other people (be they troubles of mind, body, or estate) we find almost invariably that they are only understood by the few who have passed through the same fire. These are the kind of persons who feel with us. These are the only ones who can enter thoroughly into our case, simply because their own has been similar or allied to it. You may have to suffer a good deal sometimes without meeting with any one capable of quite sympathising as you desire. Your attendants may be very kind in their way, or your fellow-sufferers may be examples of patience; but with much that ought to inspire you with cheerfulness, you are nevertheless inclined to cry out, "Ah! No one knows what I suffer." That is, "No one about me seems to realise my case, or to be able quite to comprehend its nature." So there is in the hidden depth of your heart an appeal for sympathy which no human being, however kind, seems able to respond to. Why this insatiable yearning? Is there no one who can satisfy it? Yes, One. The Son of Man, the great High Priest, is touched with sympathy in your case, is feeling with you now. Hear Him saying, "My child, I can enter as a man into the very recesses of your suffering soul. Lay its burden at My feet and leave it there. As often as you find yourself lonely in spirit, although there be many kind friends around you, come unto Me and simply ask for My sympathy, which is always yours, and I will give you rest."

